# Predicting Axillary Response in Hormone Receptor-Positive Breast Cancer after Neoadjuvant Chemotherapy Using Real-World Data

**DOI:** 10.1155/2022/6972703

**Published:** 2022-12-21

**Authors:** Jie Qiu, Yiwen Zheng, Da Qian, Dandan Guan, Qinghui Zheng, Yuhao Xu, Siyi Ju, Xuli Meng, Hongchao Tang

**Affiliations:** ^1^Second Clinical College, Zhejiang Chinese Medical University, Hangzhou 310000, China; ^2^Department of Burn and Plastic Surgery-Hand Surgery, Changshu Hospital Affiliated to Soochow University, Changshu No. 1 People's Hospital, Changshu 215500, Jiangsu, China; ^3^Department of Breast Surgery, Zhejiang Province People's Hospital, Hangzhou 310000, China

## Abstract

**Purpose:**

To develop a scoring system for hormone receptor-positive (HR+) breast cancer patients who are expected to achieve axillary pathological complete response (pCR) after neoadjuvant chemotherapy (NAC). To confirm the correlation between axillary status and survival rate in HR+ breast cancer after NAC.

**Methods:**

Women from the Shanghai Jiao Tong University Breast Cancer Database (SJTU-BCDB) who underwent NAC for cT1-4N1-3M0 primary HR+ breast cancer between 2009 and 2018 were included in the study. In this case, patient follow up was performed until 2022 for those with complete data before and after NAC. The main outcome measures were the axillary pCR rate, overall survival (OS), and disease-free survival (DFS). The patients were randomly assigned to a test set (*n* = 175) and a validation set (*n* = 68) in a 7 : 3 ratio. A prediction risk score was then developed based on the odds ratios from the multivariate analysis of the test set (*n* = 175) before being validated in the validation set (*n* = 68). Finally, the Kaplan–Meier curves were used to explore the survival on this score system.

**Results:**

From the database, 243 women were included, and the median follow-up period was 47.5 months (95% confidence interval: 41.9–53.1). The axillary pCR rate was 18.9% (46 of 243), with the independent predictors of residual positive axillary lymph nodes (LNs) being lymphovascular invasion (LVI), breast conserving surgery (BCS), Ki67 < 14%, HER2 negativity, positive lymph nodes in ultrasound (US) before surgery, and stage III histological grade (All, *P* < 0.05). Using the above predictors of the model, the receiver operating characteristic (ROC) curve was used for calibration and inspection, with values for the test and validation sets being 0.847 (*P* < 0.001; 95% CI: 0.769, 0.925) and 0.813 (*P* < 0.001; 95% CI: 0.741, 0.885), respectively. The total risk score ranged from 0 to 6 for the multivariate analysis, and from this range, a risk score of 0–2 was defined as a low-risk group, while scores of 3–6 were defined as the high-risk one. By constructing the survival curve, it was found that the 5-year OS rates for the low-risk and high-risk groups were 89.0% and 84.2% (*P* = 0.236). Similarly, the 5-year DFS rates for the low-risk and high-risk groups were 80% and 68.5% (*P* = 0.048), respectively. In addition, axillary pathological stages were significantly correlated with the overall survival (OS) and disease-free survival (DFS) (All, *P* < 0.05).

**Conclusion:**

The prediction model showed good performance for HR + breast cancer. LVI, BCS, low Ki-67, HER2 negativity, suspected positive LNs before surgery, and stage III histological grade were all risk factors for residual positive axillary LNs. However, unlike pathological stages, achieving pCR in the axillary LNs does not affect the survival status.

## 1. Introduction

Breast cancer (BC) is the most common malignancy among women, with incidence rates even higher than for lung cancer [1], and it is generally divided into triple-negative breast cancer (TNBC), human epidermal growth factor receptor 2 (HER2) positive, and hormone receptor-positive (HR+). The latter can be further classified as luminal A, luminal B HER2−, and luminal B HER2+ which tend to be highly sensitive to endocrine therapy after surgery [2, 3]. However, research on preoperative neoadjuvant chemotherapy (NAC) for these types of BC is still rarely reported. Currently, NAC is the standard treatment for patients with locally advanced or inoperable tumors, and it is frequently used to reduce tumor size, improve breast conserving surgery (BCS) rates, and reduce axillary metastasis, even in those who should have undergone axillary dissection turned to sentinel lymph node biopsy [4]. In this context, pathological complete response (pCR) is considered to be the most important indicator to evaluate the efficacy of NAC; patients who achieve pCR in breast and axillary lymph nodes (LNs) after NAC have improved survival outcomes and disease free survival times regardless of their initial status [5, 6]. However, the pCR rate varies with breast cancer subtype, being lower in HR+ compared with HER2+ and TNBC, even though the former has a better overall prognosis [7, 8]. Therefore, NAC seems to be less effective for HR+ breast cancer, with the decision to use NAC in those patients remaining controversial.

Achieving pCR is the most common target of NAC, and patients are still encouraged to perform BCS after effective NAC, including those for whom total mastectomy was initially considered as an option [9]. However, few studies have, so far, evaluated the axillary pCR rate for HR+ patients who received NAC [9, 10].

To efficiently select those HR+ patients who would benefit from NAC in the armpit, the clinical characteristics and treatment methods of cT1-4N1-3M0 patients who achieved axillary pCR after NAC were compared with those who did not. A model was subsequently developed to predict the response of axillary LNs to NAC in patients with clinical node-positive breast cancer. In this case, clinical lymph node positivity was defined as imaging positivity or punctures positivity and was evaluated by ultrasound (US), as required by the standards of the American College of Radiology (ACR) [11].

## 2. Materials and Methods

Patients with HR+ breast cancer but no distant metastasis, biopsy-confirmed ones, or those with previously untreated diseases were eligible for enrollment, with their data selected from the breast cancer database of Ruijin Hospital, Shanghai. This retrospective study was approved by the institutional review board of the authors' institution, and the need for written informed consent was waived.

### 2.1. Patient Selection

For this study, 243 patients treated with NAC prior to surgery were identified for the period between September, 2009 and December, 2018. All the patients underwent axillary ultrasounds before and after NAC. In this case, as per definition, clinically node-positive breast cancer was either pathologically confirmed or included clinically suspected axillary LNs metastasis. Axillary pCR was then defined as the absence of metastasis in surgical pathology. These patients were randomized in a 7 : 3 ratio to the test set (*n* = 175) and the validation set (*n* = 68).

**Figure 1:**
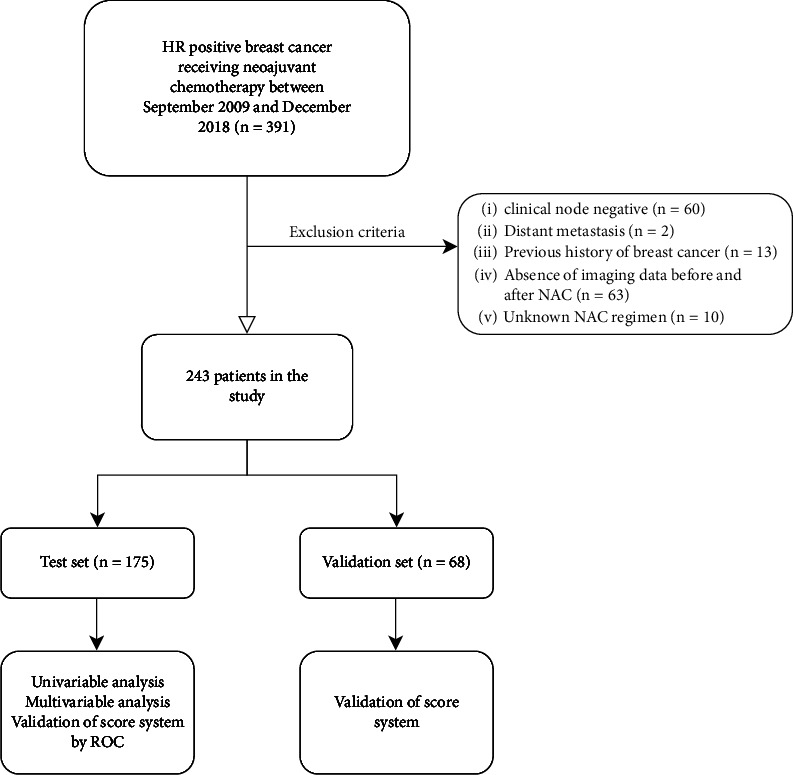
Study profile of 391 patients who received NAC and subsequently underwent surgery between September, 2009 and December, 2018. Of these, 243 patients met the eligibility criteria and were enrolled in this study. NAC = neoadjuvant chemotherapy.

Based on clinical physical examination and imaging data, the following patients were excluded: clinically node-negative patients (*n* = 60), those for whom imaging data were absent, especially axillary US before or after NAC (*n* = 63), those with distant metastasis at initial status (*n* = 2), those with previous breast cancer history (*n* = 13) and those with unknown NAC data (*n* = 10) (Figure 1). All the patients received anthracycline and taxol-based NAC as required by the National Comprehensive Cancer Network (NCCN) [12]. Of the 243 included patients, 18.93% (46 of 243) achieved axillary pCR after NAC. If the US showed suspected cases of axillary LNs before surgery, the patients underwent axillary dissection. Conversely, sentinel lymph node biopsy (SLNB) was performed in patients without suspected LNs and for whom axillary dissection would have been performed if they were positive [13].

### 2.2. Data Collection

Clinical and pathologic data, including age, body mass index (BMI), menopausal status, initial clinical and final T stage, initial clinical and final N stage, histologic type, breast surgery method, axillary surgery method, pathological T stage, and N stage were collected. In addition, information on Ki-67 expression, HER2 status, NAC regimen, and cycle and the lymphovascular conditions of all available patients was acquired. The primary variable was the axillary pCR rate for patients who had received NAC.

### 2.3. Statistical Analysis

Data were analyzed with the IBM SPSS statistics software version 25 (SPSS, Inc., Chicago, IL, USA), while GraphPad Prism version 9.0 (GraphPad Software, CA, USA) was used for generating images. The patient group was randomly assigned to either the test set or the validation set in a 7 : 3 ratio by using the random sampling method. For the test set, logistic regression analysis was then used to examine the factors associated with residual positive axillary LNs. In this case, after univariate analysis, covariates with *P* values <0.2 were included in multivariate analysis. To develop a generalized axillary correlation prediction model, each factor was defined as 1 which indicated the risk score. Hence, the total risk score for each patient in the test set was obtained from the sum of the eligible risk scores [14]. This prediction model was subsequently validated in another randomly assigned group. In this case, the Hosmer–Lemeshow goodness-of-fit test and the area under the receiver operating characteristic curve (AUC) were used to evaluate the calibration and identification, with *P* values <0.05 indicating the statistical significance of the results. Furthermore, survival curves were calculated using Kaplan–Meier analysis, while the level of significance was determined by the log rank (Mantel–Cox) test.

## 3. Results

### 3.1. Baseline Characteristics

Overall, 243 HR+ breast cancer patients were included in the study (Figure 1), with their clinical and pathological features before and after NAC listed in Table 1. These patients, with a mean age and BMI of 50.24 years ± 11.1 and 24.2 ± 3.5, respectively, were further divided into two groups, namely, a test set of 175 patients and a verification set of 68 (7 : 3 ratio). Among these patients, 62.55% (152 of 243) had pathological axillary metastasis based on core-needle biopsy. Furthermore, the percentage of patients with clinical T1, T2, T3 and T4 tumors were 13.6%, 59.7%, 13.2%, and 13.6%, respectively, with the most common histological type being invasive ductal carcinoma (91.4%, 222 of 243). After NAC, 154 patients (63.4%) underwent total mastectomy, while 89 underwent breast-conserving surgery (36.6%). As far as axillary treatment was concerned, only 1.2% (3 out of 243) underwent SLNB, with ALND performed for the remaining 98.8% (240 of 243) of the patients. Finally, for primary tumor, pCR was observed in 6.6% (16 of 243) of patients, while for axillary nodes, the proportion was 18.9% (46 of 243). Thus, it was clear that the pCR rate achieved by NAC in HR+ breast cancer was not ideal for both tumor and axillary sites. There was also no statistical difference in the distribution of the variables between the test and validation sets (all, *P* > 0.05).

After surgery, 34.6% (84 of 243) of the patients underwent chemotherapy again, 78.6% (191 of 243) received conventional adjuvant radiotherapy, and 87.2% (212 of 243) received endocrine therapy. Follow up was performed for all patients, with a median period of 47.5 months.

### 3.2. Factors and Prediction of Residual Positive Axillary LNs

In the test set (*n* = 175), 20.6% (36 of 175) of the patients achieved axillary pCR, and 79.4% (139 of 175) had residual positive axillary LNs (Table 1).  Table 2 compares the axillary pCR and residual positive axillary LNs in this set (*n* = 175). Univariate analysis first showed that the clinical N2 stage was more common in residual positive axillary LNs compared with axillary pCR (*P* < 0.2). Moreover, after completing neoadjuvant chemotherapy, patients with residual positive axillary LNs were more likely to present suspected nodules by US before surgery (*P* < 0.2). Finally, pathological examinations suggested that lower Ki-67, HER2 negative, histological III grade and lymphovascular invasion were significantly associated with residual positive axillary LNs (*P* < 0.2). Overall, no significant differences in age, clinical T stage, histologic type and grade, axillary surgery, as well as NAC regimens and cycles, were noted between the two groups (All, *P* > 0.2).

From multivariate analysis (Table 3), LVI (OR, 6.438; 95% CI:1.715–24.168), BCS (OR, 4.972; 95% CI:1.398–17.688), HER2 negativity (OR, 3.117; 95% CI:1.135–8.562), low Ki-67 (OR, 3.671; 95% CI:1.284–10.495), suspected positive axillary before surgery (OR, 4.613; 95% CI:1.495–14.238) and histological grade III (OR, 4.809; 95% CI:1.332–17.366) (All, *P* < 0.05) were identified as independent predictors of residual positive axillary LNs (Tables 2 and 3).

**Figure 2:**
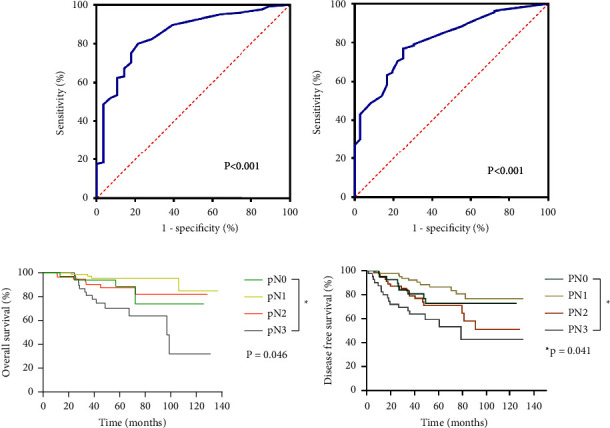
(a) The AUC in the test set for the risk score based on the ROC curves, *P* < 0.001. (b) The AUC of the validation set for the risk score based on the ROC curves, *P* < 0.001. (c) Overall survival according to the pN stage after neoadjuvant chemotherapy (NAC), *P* = 0.046. (d) Disease free survival to pN stage after NAC, *P* = 0.041.

The predictive model was then established based on each of the above predictor. In this case, each predictor had a risk score of 1, with the total risk score ranging from 0 to 6. ROC was subsequently used to show that the prediction model had good recognition (AUC, 0.847; 95% CI: 0.769, 0.925) and correction abilities (Hosmer–Lemeshow test validity, 0.556; *P* < 0.001). In addition, after performing internal validation, the AUC was found to be 0.813, (95% CI: 0.741, 0.885), which was indicative of good calibration ability (Hosmer–Lemeshow test validity, 0.937; *P* < 0.001) (Figures 2(a), 2(b)). For the test and the validation sets, the higher the total risk scores, the greater the probability of having residual positive axillary LNs.

### 3.3. Stratification of Survival Time by Axillary Pathological Status

Survival curves for DFS and OS, based on the axillary pathological nodal status after NAC, are shown in Figure 2. This group of patients, for which the axillary pCR rate was low, had a 5-year OS rate of 88.6% versus 87.1% for pN0 versus pN+ (*P* = 0.824) as well as a 5-year DFS rate of 72.8% versus 75.8% for pN0 versus pN+ (*P* = 0.804). Furthermore, there was no correlation between lymph nodes status and survival time after NAC (Figure S1). Interestingly, results of axillary staging after NAC could predict survival. This not only confirms the prognostic value of axillary pathological status after NAC but also supports the importance of surgical axillary staging in this group. The survival time of the 243 patients is also shown in Figures 2(c) and 2(d). In this case, the values for axillary pN0, pN1, pN2, and pN3 were 46 (18.9%), 95 (39.1%), 60 (24.7%), and 42 (17.3%), respectively, while the 5-year OS rate was 88.6%, 95.4%, 87.5%, and 70.3%, respectively. In particular, the OS was significantly different between these groups (*P* < 0.05). Regarding the 5-year DFS rate, the values were pN0 (76.3%), pN1 (86.3%), pN2 (71.7%), and pN3 (59.2%), with the results being also significantly different between the groups (*P* < 0.05).

**Figure 3:**
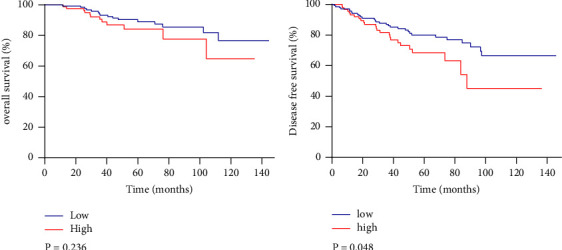
(a) OS rate of the low-risk and high-risk groups, *P* = 0236. (b) DFS rate of low-risk and high-risk groups, *P* = 0.048.

Based on the data in Table 3, if patients meet one of the criteria, the risk score could be simplified to 1, with a median of 3 then used as the dividing line. As such, 0 to 2 was defined as a low-risk group, while 3 to 6 was considered as the high-risk one. New survival curves were then generated based on the above thresholds (Figures 3(a) and 3(b)). In this case, the 5-year OS rate in the low-risk group was 87.5%, and that of the high-risk group was 86.7%. Similarly, the corresponding 5-year DFS for the low-risk and high-risk groups were 78.5% and 70.3%, respectively. Altogether, the results showed that, after NAC, the risk score of axillary LNs was statistically significant with the DFS.

## 4. Discussion

In our study, among HR+ breast cancer patients who received NAC, the overall breast pCR rate was 6.6%, while the axillary one was 18.9%. Therefore, the results showed that the pCR rate in HR+ patients after NAC can be unsatisfactory, and this was consistent with previous studies [15, 16]. The current study analyzed preoperative factors associated with axillary pathological status to establish a model that could predict whether HR+ breast cancer tends to achieve axillary pathological remission. This prediction model successfully evaluated the axillary response after NAC. Through univariate and multivariate analysis, six risk factors responsible for a poor effect in the armpit after NAC were identified. First, US, which provides imaging evidence of axillary lymph nodes, remain the most common method for evaluating axillary response after NAC. In addition, LVI is associated with distant metastasis in various solid tumors and is an adverse prognostic marker of survival and recurrence [17]. In previous studies, it only existed in surgical pathology reports, and its molecular biology is poorly understood. It is used to evaluate the survival prognosis of early gastric cancer in the past, patients with positive LVI should be considered as candidates for adjuvant chemotherapy [18, 19]. Similarly, the study has found that LVI is an independent predictor of survival in breast cancer after neoadjuvant chemotherapy, some scholars also have found that the occurrence of LVI is significantly related to luminal B with HER2(-), and the basal-like subtype [20]. Cheung SM and Bo Bae Choi et al. considered that preoperative magnetic resonance imaging (MRI) and diffusion-weighted imaging (DWI) can help diagnose the existence of LVI in breast cancer [21, 22]. Actually, in our multivariate analysis about HR+ population, patients with LVI had the strongest independent association with residual positive axillary LNs (OR, 6.438; *P* < 0.05). We expect that LVI will become a better marker for predicting recurrence and prognosis in the future. To sum up, tumor biology has been an important factor in predicting the pathological response in both breast and the armpit. In this study, for HER2-negative patients with histological grade III tumors and low expression of Ki-67, axillary pCR was not easily obtained, and this was consistent with previous research findings [15]. Surprisingly, regardless of chemotherapy regimens or cycles, it did not influence axillary pCR. To summarize, our imaging-based and pathology-based predictive model could fairly discriminate between axillary pCR and residual positive axillary LNs. At the same time, AUC values of 0.847 and 0.813 for the test and validation groups showed that the model was accurate and effective.

Based on multivariate analysis, we summarized the above factors as risk factors of axillary pCR with 1 point for each index, and patients with risk scores of 0 to 2 were defined as the low-risk group while 3–6 was considered to be the high-risk one. The low-risk group actually had 29.6% (42/142) of pCR rate while the high-risk one had a 4.0% (4/101) pCR rate; it shows the low and high defined scores of our model are ideal. On the survival analysis of the Kaplan–Meier curve, it was further found that the lymph node status after NAC was not associated with OS, but instead was related to DFS (*P* = 0.048). Although the pathological stage was significantly related to survival (*P* < 0.05), it was speculated that this could be related to an insufficient number of patients as well as variations in the treatment received after the operation.

Current methods for the clinical evaluation of axillary LNs usually include physical examination and US. Some scholars believed that lymph node staging before NAC allowed local treatment decisions to be made without the risk of undertreatment [23]. However, the number of patients with clinical positive LNs and who underwent axillary biopsy was not high. Studies have shown that the sensitivity of needle biopsy was only 25% [24], with approximately 50% of women with axillary invasion identified preoperatively [25]. Even though some patients actually did achieve axillary pCR after NAC, ALND was still performed, the survival rate was not significantly different compared with residual positive axillary LNs (*P* > 0.05). Unnecessary ALND increases the scope of axillary damage in patients, leading to complications after surgery, such as lymphedema and muscle strength decline. Hence, our model can guide management and treatment in the armpit so as to avoid unnecessary radiation therapy and complications.

We further reflected on the limitations and shortcomings of the model. First, ultrasound is an important measure for imaging evaluation, but we found that in suspected cases of positive axillary LNs by US, benign lymphadenopathy cannot be ruled out. The false negative rate (FNR) has been a difficult problem for many scholars, in previous studies; the FNR was about 5–15% [11, 26]. Although we have the diagnosis of ultrasound intervention experts with senior professional titles, we still cannot avoid the phenomenon of false negativity. Therefore, an MRI of the breast and armpit is necessary for those who cannot be punctured but have suspicious lymph nodes under ultrasound. Second, based on the data in this article, the axillary pCR rate is 18.9% (46/243), and the breast pCR rate is 6.6% (16/243). Among 46 pN0 patients, 13 cases both achieved breast pCR. On the contrary, most of the patients who reached pT0 achieved pN0 (13/16). From here, we can see that our model is only suitable for evaluating axillary pCR, not breast pCR. Third, HER2 shown by preoperative puncture may not be consistent with postoperative pathology due to the heterogeneity of tumors. Finally, the number of patients in our study was quite low, and it is only a retrospective study; and we are expected to include it in the prospective study.

In conclusion, this study's predictive model based on imaging and pathology can help doctors and patients assess the responsiveness of HR+ breast cancer to NAC, especially in those with clinically positive LNs. As such, the model can guide the management of axillary LNs and avoid unnecessary dissection.

## Figures and Tables

**Table 1:** Baseline characteristics of patients.

Characteristics	Total patients (*n* = 243)	Test sets (*n* = 175)	Validation sets (*n* = 68)	*P* values
Age (years)	50.24 ± 11.1	51.1 ± 10.3	49.4 ± 12.3	0.257
BMI	24.2 ± 3.5	24.4 ± 4.5	24.0 ± 3.5	0.664
Menopausal status				0.394
Premenopausal	118	82/46.9%	36/52.9%	
Postmenopausal	125	93/53.1%	32/47.1%	
Clinical T stage				0.705
1	33	26/14.9%	7/10.3%	
2	145	105/60.0%	40/58.8%	
3	32	22/12.6%	10/14.7%	
4	33	22/12.6%	11/16.2%	
Clinical N stage				0.598
1	128	95/54.3%	33/48.5%	
2	81	55/31.4%	26/38.2%	
3	34	25/14.3%	9/13.2%	
Histologic type				0.998
IDC	222	160/91.4%	62/91.2%	
ILC	7	5/2.9%	2/2.9%	
Other	14	10/5.7%	4/5.9%	
Breast surgery				0.389
BCS	89	67/38.3%	22/32.4%	
Mastectomy	154	108/61.7%	46/67.6%	
LN surgery				0.835
SLNB	3	2/1.1%	1/1.5%	
ALND	240	173/98.9%	67/98.5%	
ypT stage				0.455
0	16	12/6.9%	4/5.9%	
1	122	90/51.4%	32/47.1%	
2	82	59/33.7%	23/33.8%	
3	14	10/5.7%	4/5.9%	
4	9	4/2.3%	5/7.4%	
ypN stage				0.348
0	46	36/20.6%	10/14.7%	
1	95	68/38.9%	27/39.7%	
2	60	45/25.7%	15/22.1%	
3	42	26/14.9%	16/23.5%	

*Note.* Unless otherwise stated, data refer to the number of patients or the percentage of the total number of patients. IDC = invasive ductal carcinoma; ILC = invasive lobular carcinoma; BCS = breast conserving surgery; SLNB = sentinel lymph node biopsy; ALND = axillary lymph node dissection.

**Table 2:** Univariate analysis of axillary in patients from the test set (*n* = 175).

Characteristics	OR	95% Cl	*P* values
Age	0.990	0.956–1.027	0.602
Clinical T stage			
1	REF		
2	0.552	0.174–1.751	0.313
3	1.818	0.300–11.023	0.516
4	0.818	0.179–3.739	0.796
Clinical N stage			
1	REF		
2	2.723	1.096–6.762	0.031
3	4.566	1.007–20.714	0.049
US of lymph nodes			
Negative	REF		
Positive	4.102	17.47–9.631	0.001
Histologic type			
IDC	REF		
Other	0.570	0.123–2.649	0.473
NAC regimes			
A	REF		
T	0.455	0.084–2.469	0.361
A + T	1.131	0.418–3.062	0.808
NAC cycles			
<4	REF		
4–6	0.553	0.063–4.844	0.593
<6	0.769	0.085–6.944	0.815
Breast surgery			
BCS	4.992	1.833–13.598	0.002
Mastectomy	REF		
ypT stage			
0	REF		
1–4	1.806	0.523–6.237	0.350
Histologic grade^*∗*^			
I/II	REF		
III	2.211	0.828–5.904	0.113
Ki-67			
<14	3.346	1.544–7.252	0.002
≥14	REF		
HER2^*∗*^			
Negative	2.367	1.011–5.538	0.047
positive	REF		
LVI			
Negative	REF		
Positive	3.634	1.329–9.937	0.012

OR = odds ratio; CI = confidence interval; REF = reference; HER2 = human epidermal growth factor receptor 2; LVI = lymphovascular invasion. ^*∗*^Only patients with available data were used in the analysis.

**Table 3:** Multivariate regression analysis for the test set (*n* = 175).

Characteristics	Odds ratios	95% CI	*P* values
LVI (positive)	6.438	1.715–24.168	0.006
Breast surgery (BCS)	4.972	1.398–17.688	0.013
HER2 (negative)^*∗*^	3.117	1.135–8.562	0.027
Ki-67 (<14%)	3.671	1.284–10.495	0.015
US of lymph nodes (positive)	4.613	1.495–14.238	0.008
Histologic grade (III)^*∗*^	4.809	1.332–17.366	0.017

CI = confidence interval; LVI = lymphovascular invasion; BCS = breast conserving surgery; HER2 = human epidermal growth factor receptor 2; US = ultrasound. ^*∗*^Only patients with available data are used in the analysis.

## Data Availability

The data used to support the findings of this study are included within the article.

## Supplementary Materials

Supplementary Materials Figure S1: A. Overall survival based on axillary nodal status after neoadjuvant chemotherapy (NAC), *P* = 0.824; B. Disease free survival to axillary nodal status after NAC, *P* = 0.804. pN0 means axillary lymph nodes for which pathological complete remission (pCR) was achieved. pN+ means non-pCR.
