# Supplementary material for: Predicting Axillary Response in Hormone Receptor-Positive Breast Cancer after Neoadjuvant Chemotherapy Using Real-World Data
Source: J Oncol. 2022 Dec 21;2022:6972703. doi: 10.1155/2022/6972703 (PMC9797309; doi:10.1155/2022/6972703)
Supplement: Supplementary Materials — Figure S1: A. Overall survival based on axillary nodal status after neoadjuvant chemotherapy (NAC), P = 0.824; B. Disease free survival to axillary nodal status after NAC, P = 0.804. pN0 means axillary lymph nodes for which pathological complete remission (pCR) was achieved. pN+ means non-pCR. [file 6972703.f1.pdf]

A

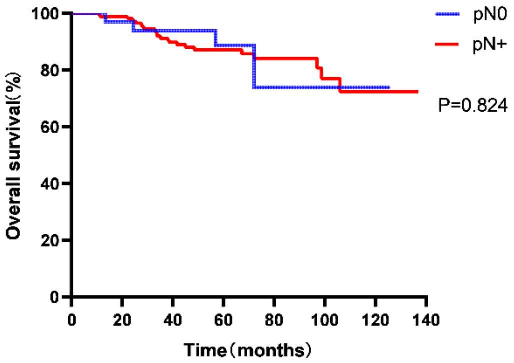

B

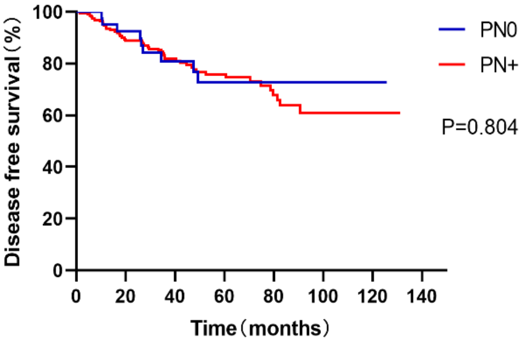

Figure S1: **A**. Overall survival according to axillary nodal status after neoadjuvant chemotherapy (NAC), P=0.824; **B**. Disease free survival to axillary nodal status after NAC, P=0.804. pN0 means axillary lymph nodes are pathological complete remission (pCR). pN+ means non-pCR.
